# Dioxazolones as electrophilic amide sources in copper-catalyzed and -mediated transformations

**DOI:** 10.3762/bjoc.21.12

**Published:** 2025-01-22

**Authors:** Seungmin Lee, Minsuk Kim, Hyewon Han, Jongwoo Son

**Affiliations:** 1 Department of Chemistry, Dong-A University, Busan 49315, South Korea; 2 Department of Chemical Engineering (BK21 FOUR Graduate Program), Dong-A University, Busan 49315, South Korea

**Keywords:** amidation, copper salts, dioxazolones, electrophilic nitrogen, *N*-acyl nitrene

## Abstract

Over the past decade, dioxazolones have been widely used as *N*-acylamide sources in amidation processes of challenging substrates, typically employing precious transition metals. However, these catalytic systems often present several challenges associated with cost, toxicity, stability, and recyclability. Among the 3d transition metals, copper catalysts have been gaining increasing attention owing to their abundance, cost-effectiveness, and sustainability. Recently, these catalytic systems have been applied to the chemical transformation of dioxazolones, conferring a convenient protocol towards amidated products. This review highlights recent advancements in the synthetic transformations of dioxazolones, with particular examples of copper salts.

## Introduction

**Scheme 1:**
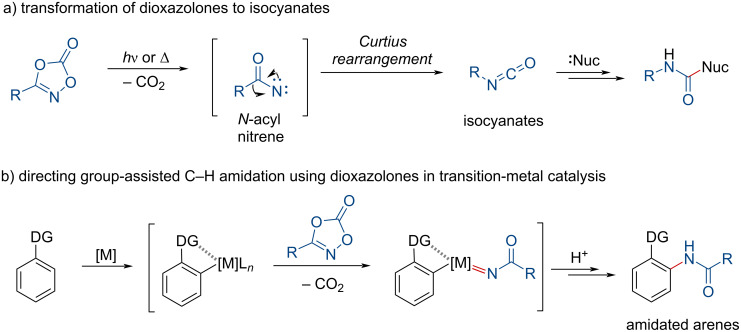
Formation of isocyanates and amidated arenes from dioxazolones.

Dioxazolones, first synthesized and reported by Beck and co-workers [1], have been employed as electrophiles in various nucleophilic transformations due to their susceptibility to rapid decomposition into the corresponding isocyanates (Scheme 1a) [2–3]. They have attracted increasing interest as electrophilic amide sources in amidation using transition-metal catalysts such as ruthenium, rhodium, and iridium (Scheme 1b) [4–19]. Notably, dioxazolones have primarily been studied in directed carbon–hydrogen amidation processes, which can circumvent the need for tedious prefunctionalizations.

Copper catalysts have gained recognition and attracted increasing interest as affordable, versatile, and sustainable catalytic systems. These catalysts are extensively employed in organic synthesis owing to their cost-effectiveness, reduced toxicity, and natural abundance [20–28]. The use of copper salts has enabled a variety of conventionally challenging functionalizations, such as *N*- or *O*-arylations using aryl halides [29–38] or arylboronic acids [39–42], hydrofunctionalizations of unsaturated motifs [25,43–56], the oxidation of alcohols [57–61], and photoinduced alkylations of various nucleophiles [22,62–68]. Recently, these sustainable catalytic systems have gradually been applied to amidations employing dioxazolones as amide sources.

To the best of our knowledge, no review article has yet covered the recent progress in the chemical transformation of dioxazolones using copper salts. This review provides an overview of the recent achievements in the use of copper salts as sustainable metal systems for the transformation of dioxazolones. This review also discusses several related proposed mechanisms.

## Review

### 1 Transformations via the formation of copper nitrenoids

#### 1.1 C(sp^3^)–H amidation

Lactams are recognized as one of the most significant nitrogen-containing heterocycles in drug discovery [69–70]. Among these, six-membered lactams, known as 2-piperidinones, have been extensively studied due to their potential bioactivity [70–73]. Despite the development of synthetic approaches for six-membered lactams, including transition-metal-catalyzed transformations, several limitations remain, particularly with regard to regioselectivity and asymmetric C–N bond formation, which are still limited. In 2023, the Chang group elegantly unveiled a protocol for an enantioselective C–N bond formation, introducing δ-lactams from dioxazolones using a copper(I) catalyst and a chiral BOX ligand [74].

**Scheme 2:**
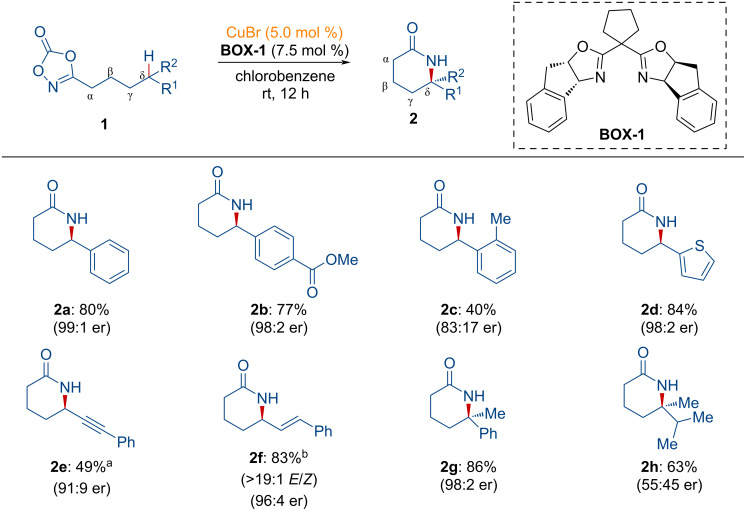
Copper-catalyzed synthesis of δ-lactams via open-shell copper nitrenoid transfer. ^a^CuBr (10 mol %) and **BOX-1** (15 mol %) were used. ^b^A stereoisomeric mixture (*E*/*Z* = 1.4:1) of dioxazolones was used.

As shown in Scheme 2, dioxazolones containing aryl and heteroaryl groups were converted into the corresponding lactams in high yields and excellent enantioselectivities (**2a**, **2b**, and **2d**). It was observed that the steric environment affected both reactivity and enantioselectivity (**2c**). Six-membered lactams featuring propargylic (**2e**) and alkenyl (**2f**) motifs were also obtained with excellent regioselectivity and enantioselectivity. Furthermore, a lactam containing a quaternary carbon center (**2g**) was prepared. However, a lower enantioselectivity was observed for product **2h** due to the similar steric environment of the two alkyl substituents.

**Figure 1:**
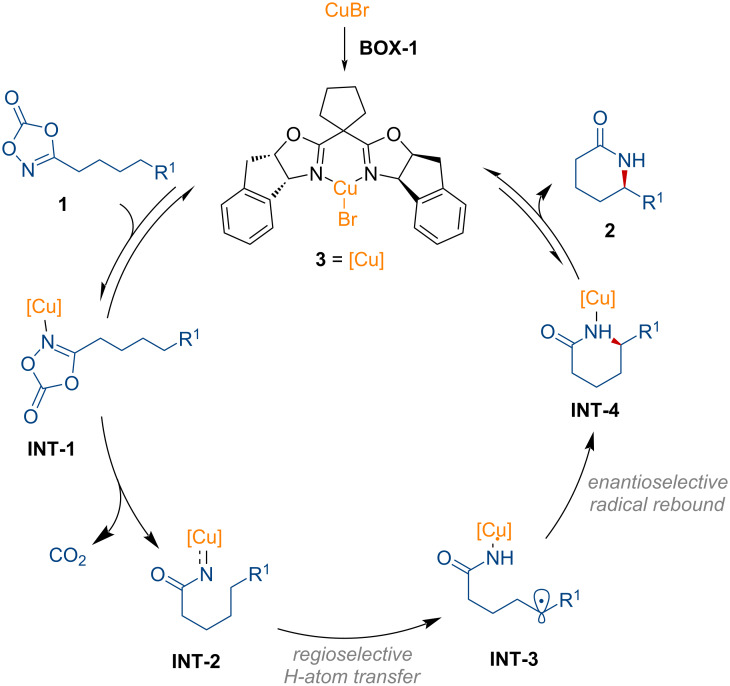
Proposed reaction pathway for the copper-catalyzed synthesis of δ-lactams from dioxazolones.

As shown in Figure 1, a catalytic cycle was proposed for the intramolecular C–H amidation process of dioxazolones. Dioxazolone **1** binds to the chiral copper complex **3**, generating the adduct **INT-1**. Decarboxylation then occurs, forming the copper nitrenoid intermediate **INT-2**, subsequently undergoing hydrogen atom transfer in a regioselective manner to afford **INT-3**. The related acyl nitrenoid intermediate was characterized by the same group [75]. Further radical rebound from **INT-4** induces the enantioselective C–N bond formation. Finally, the desired product **2** is released from **INT-4**, regenerating the active chiral copper species to participate in the catalytic cycle.

#### 1.2 C(sp^2^)–H amidation

Recently, Cao and co-workers reported the copper-catalyzed synthesis of 1,2,4-triazole derivatives via an *N*-acyl nitrene intermediate [76].

**Scheme 3:**
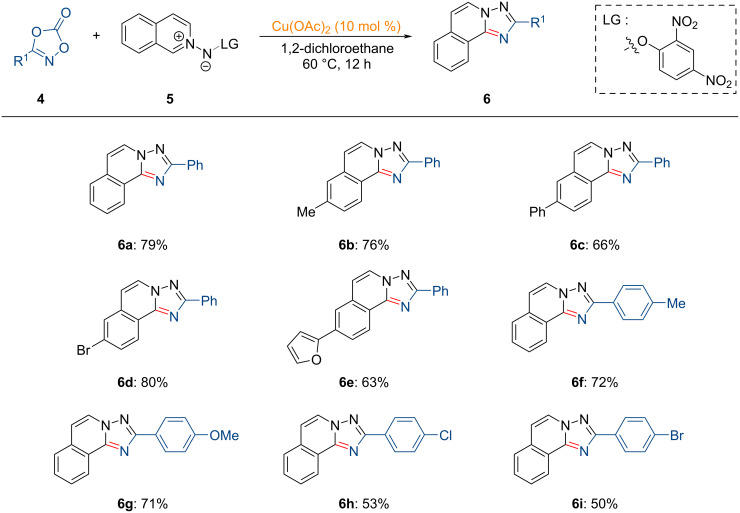
Copper(II)-catalyzed synthesis of 1,2,4-triazole derivatives.

As illustrated in Scheme 3, dioxazolones **4** and *N*-iminoquinolinium ylides **5** served as reactive substrates, leading to the formation of various polycyclic 1,2,4-triazole analogues **6**. Both dioxazolones **4** and *N*-iminoquinolinium ylides **5** demonstrated excellent tolerance in this transformation. Notably, electron-rich dioxazolones exhibited slightly higher reactivity.

**Figure 2:**
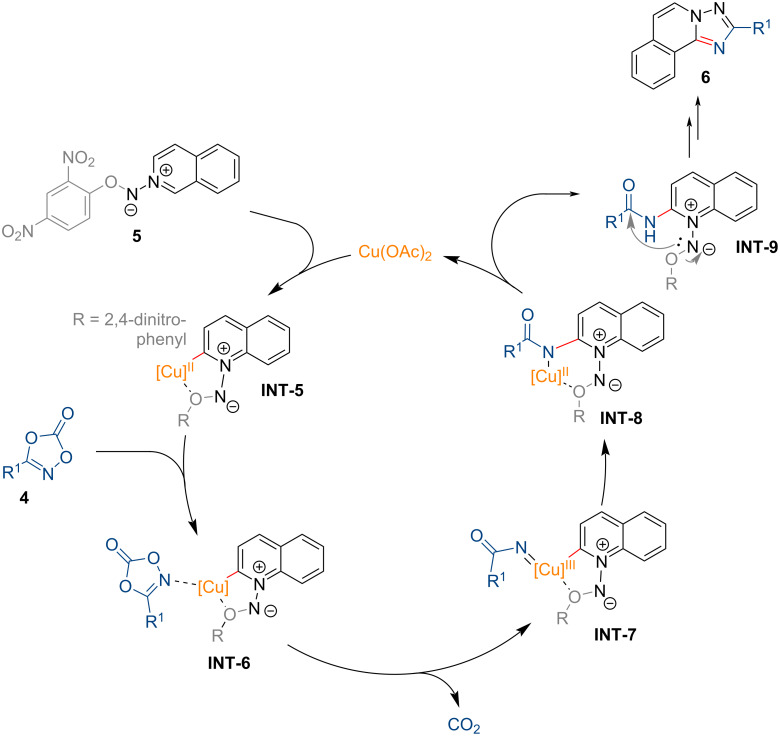
Proposed reaction mechanism for the copper-catalyzed synthesis of 1,2,4-triazole analogues from dioxazolones and *N*-iminoquinolinium ylides.

The proposed catalytic cycle for the copper-catalyzed synthesis of 1,2,4-triazole derivatives is depicted in Figure 2. The reaction is initiated by formation of the five-membered copper-containing intermediate **INT-5** through coordination of Cu(OAc)_2_ with the *N*-iminoquinolinium ylide species **5**. This process is followed by decarboxylative N–O bond insertion into **4**, yielding the *N*-acyl copper(III) nitrenoid intermediate **INT-7**. Subsequent nitrene insertion, protodemetalation, and intramolecular cyclization furnish the desired 1,2,4-triazole.

#### 1.3 Three-component formation of *N*-acyl amidines

In 2019, *N*-acyl amidines were prepared from dioxazolones using a copper catalyst with terminal alkynes and secondary amines via an *N*-acyl nitrene intermediate [77]. Amidines, found in biologically active compounds, have been widely investigated in medicinal chemistry due to their potent antiviral, antibacterial, anticancer, and other therapeutic properties [78–81].

**Scheme 4:**
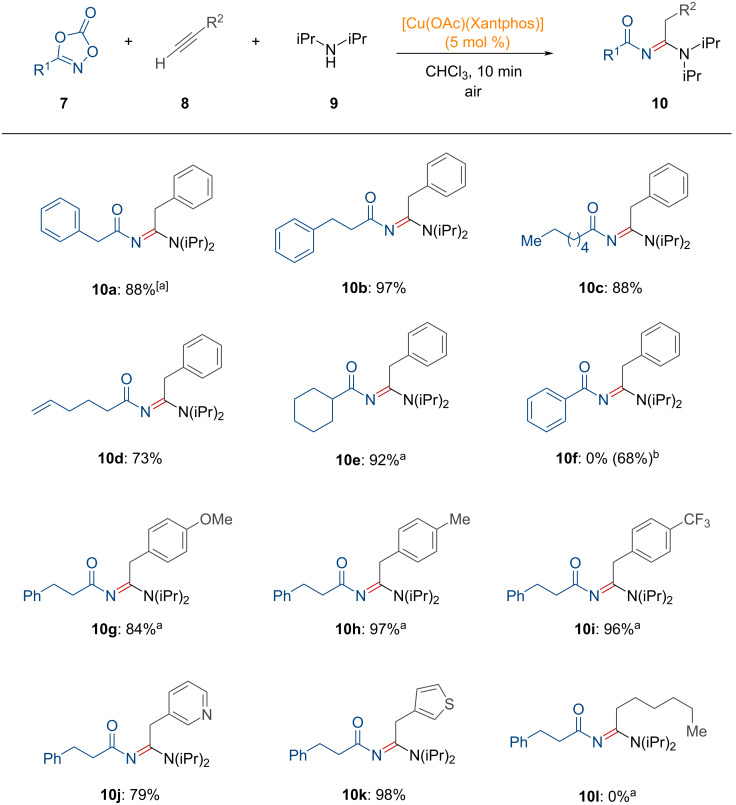
Copper(I)-catalyzed synthesis of *N*-acyl amidines from dioxazolones, acetylenes, and amines. ^a^Performed under N_2_ atmosphere. ^b^10 mol % of CuI was used instead of [Cu(OAc)(Xantphos)], with a reaction time of 30 min.

**Figure 3:**
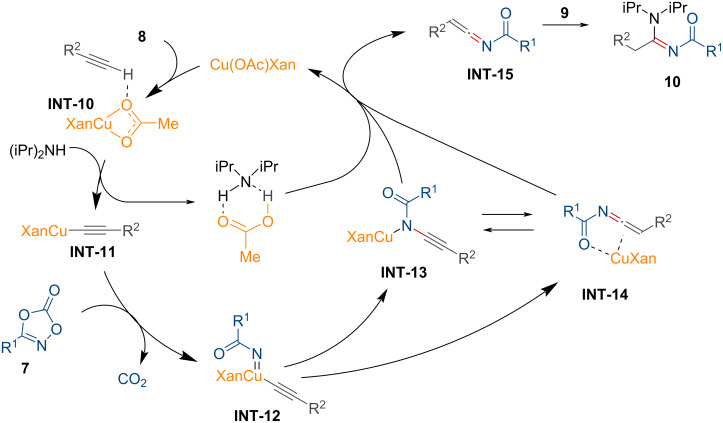
Proposed reaction mechanism for the copper(I)-catalyzed synthesis of *N*-acyl amidines.

As shown in Scheme 4, dioxazolones bearing linear alkyl groups were transformed into *N*-acyl amidines **10a**–**c** by copper catalysis. Moreover, good functional group tolerance was observed with a terminal alkene motif (**10d**). The cyclohexyl-substituted dioxazolone successfully provided the corresponding *N*-acyl amidine **10e**. However, the dioxazolone bearing a phenyl group showed no reactivity toward benzoyl amidine under the optimized reaction conditions. Instead, the authors employed a less bulky copper iodide catalyst in the absence of phosphine, successfully affording aryloyl amidine **10f**. Furthermore, the electron-rich ethynylanisole afforded the product **10g** in good yield. Acetylenes containing tolyl and trifluorophenyl substituents also exhibited improved reactivity (**10h** and **10i**). Heteroaromatic acetylenes were effective in this transformation, forming *N*-acyl amidines **10j** and **10k**, respectively. On the other hand, the use of linear terminal alkynes did not result in the desired *N*-acyl amidine **10l**. Based on the substrate scope of acetylenes, the authors noted that the lower acidity of terminal acetylenes led to a diminished formation of the copper acetylide intermediate. Based on several mechanistic experiments and density functional theory (DFT) calculations, a proposed reaction mechanism is suggested as shown in Figure 3.

Initially, copper acetylide **INT-11** is formed by the reaction of acetylene with a copper precatalyst, leading to the formation of an *N*-acyl nitrene acetylide intermediate **INT-12** after the incorporation of dioxazolone **7**. Subsequently, nitrene insertion of **INT-12** into the Cu–C bond, forms **INT-13**, which then undergoes isomerization and protodemetalation, followed by catalyst regeneration, as suggested by the DFT calculations. Finally, the nucleophilic addition of the amine to the electrophilic intermediate **INT-15** leads to the formation of the *N*-acyl amidine product **10**.

#### 1.4 *N*-Arylation of dioxazolones

**Scheme 5:**
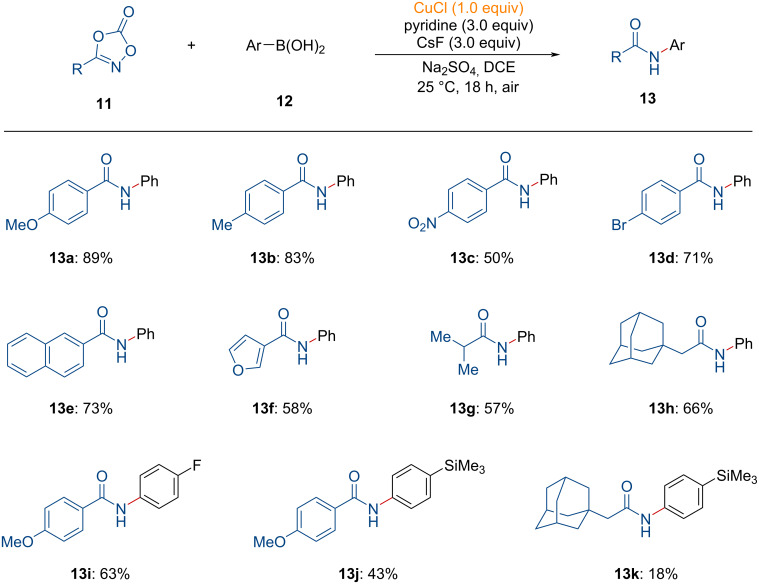
Preparation of *N*-arylamides from dioxazolones and boronic acids using a copper salt.

Amides bearing *N*-substituents are key structural motifs in a wide range of polymers [82], natural products [83], and pharmaceuticals [84–85]. Conventional synthetic routes for *N*-arylamides typically involve the condensation of carboxylic acids with anilines, often promoted by activating agents such as thionyl chloride or peptide coupling agents [86–89]. However, these protocols rely on environmentally harmful reagents and produce unwanted byproduct wastes. Recently, the research group of Son showcased a synthetic methodology for *N*-arylamides **13** from dioxazolones **11** and boronic acids **12** using copper salts (Scheme 5) [90].

**Figure 4:**
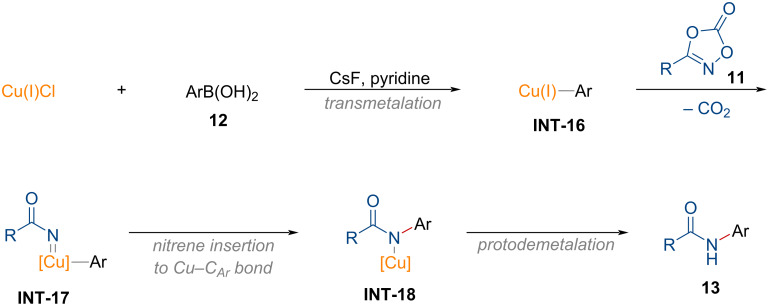
Proposed reaction pathway for the copper-mediated synthesis of *N*-arylamides from dioxazolones.

As illustrated, dioxazolones with both electron-rich and electron-poor substituents, including alkyl substituents, were well-tolerated, providing the desired products **13a**–**h**. However, the substrate scope of boronic acids was limited in this transformation. To elucidate the reaction process, kinetic and control experiments were conducted, which led to the proposed reaction pathway for the copper-mediated synthesis of *N*-arylamides from dioxazolones as shown in Figure 4.

Initially, copper(I) chloride reacts with boronic acids, forming the copper aryl complex **INT-16**, which then undergoes decarboxylative N–O bond insertion to generate the copper nitrenoid intermediate **INT-17**. Thereafter, nitrene insertion into the copper–carbon bond occurs, forming a new C(sp^2^)–N bond (**INT-18**). Finally, the desired *N*-arylamide **13** is yielded through protodemetalation. In this process, copper does not undergo further catalytic turnover, presumably due to the formation of inactive copper fluoride or copper hydroxide species.

#### 1.5 *N*-Phosphorylation of dioxazolones

**Scheme 6:**
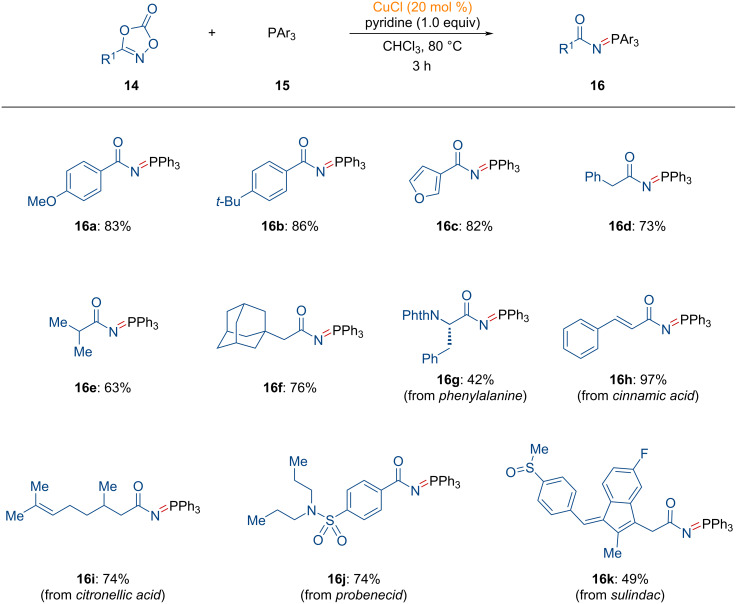
Copper-catalyzed preparation of *N*-acyl iminophosphoranes from dioxazolones.

Recently, Son and Kuniyil reported the *N*-phosphorylation of dioxazolones using organic phosphines and copper catalysts [91]. As shown in Scheme 6, a variety of dioxazolones **14** were explored for the synthesis of *N*-acyl iminophosphoranes **16**. Dioxazolones containing aryl and heteroaryl groups were successfully transformed into the desired products in high yields (**16a**–**c**).

Moreover, alkyl groups were tolerated during the formation of *N*-acyl iminophosphoranes (**16d**, **16f**). It is noteworthy that dioxazolones derived from bioactive motifs, such as peptides, natural products, and commercially available drugs were also compatible with this transformation, providing the corresponding products (**16g**–**k**).

**Figure 5:**
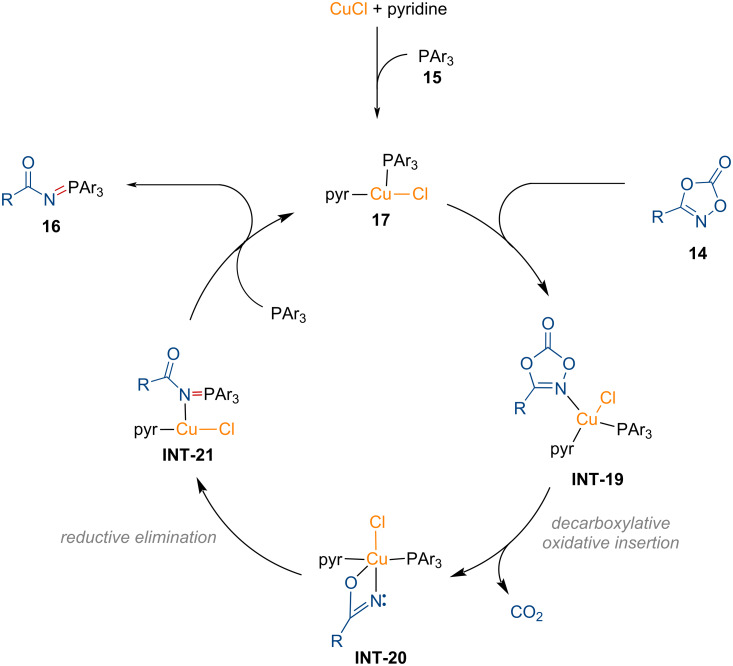
Proposed reaction pathway for the copper-catalyzed synthesis of *N*-acyl iminophosphoranes from dioxazolones.

Mechanistically, the reaction begins with the generation of the active copper species **17**, successively forming **INT-19** and **INT-20** (Figure 5). The penta-coordinated copper nitrenoid species **INT-20**, as suggested by DFT calculations, undergoes reductive elimination to yield the stable intermediate **INT-21**. This intermediate releases the *N*-acyl iminophosphorane **16** through the incorporation of another organic phosphine, thereby regenerating the active copper species **17**.

#### 1.6 Synthesis of *N*-sulfenamides

**Scheme 7:**
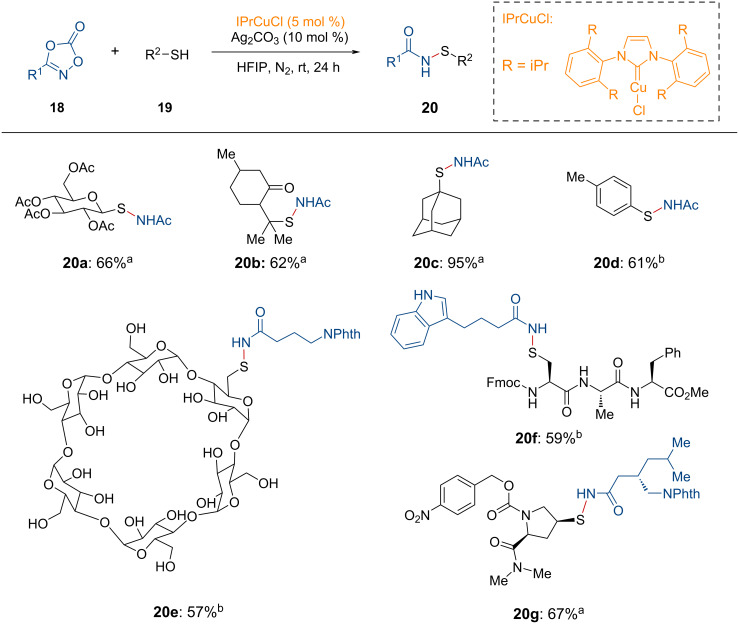
Copper-catalyzed synthesis of *N*-acyl sulfenamides. ^a^1.0 equiv of **18** and 2.0 equiv of **19** were used. ^b^2.0 equiv of **18** and 1.0 equiv of **19** were used.

In 2022, the research groups of Wang and Chen introduced a modular copper-catalyzed method for the synthesis of *N*-acyl sulfenamides **20** from dioxazolones **18** using thiols **19** via nitrogen–sulfur bond formation (Scheme 7) [92]. Secondary and tertiary thiols were highly effective in affording the corresponding *N*-acyl sulfenamides **20a**–**d**. Moreover, the bioactive motifs on both thiols and dioxazolones were well tolerated in late-stage functionalizations, representing excellent chemoselectivity (**20e**–**g**). This copper-catalyzed conjugative strategy allows for the modular preparation of biologically relevant *N*-acyl sulfenamides.

**Figure 6:**
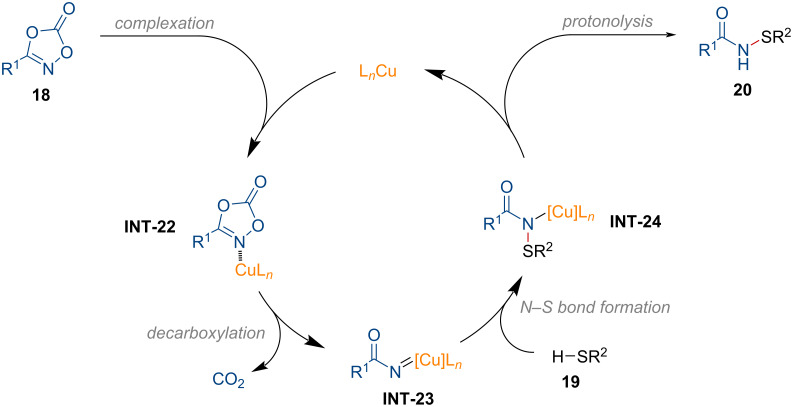
Proposed reaction mechanism for the copper-catalyzed *S*-amidation of thiols.

Based on several mechanistic experiments, a plausible reaction pathway is described in Figure 6. Decarboxylation of dioxazolone in **INT-22** forms the copper nitrene intermediate **INT-23**. The thiol then attacks the electrophilic nitrogen center of **INT-23**, which further leads to the formation of intermediate **INT-24**. Finally, the desired product **20** is afforded through protonolysis, regenerating the active copper species to complete the catalytic cycle.

### 2 Amidation via oxidative insertion to N–O bonds and reductive elimination

#### 2.1 Hydroamidation of vinylarenes

Amines bearing stereogenic centers have been widely investigated in the research area of medicinal chemistry [93–97]. In 2018, Buchwald and co-workers unveiled the enantioselective synthesis of benzylic amines through the asymmetric Markovnikov hydroamidation of alkenes utilizing diphenylsilane in copper catalysis under mild reaction conditions [98]. Dioxazolones, as amide sources, were employed in this transformation.

**Scheme 8:**
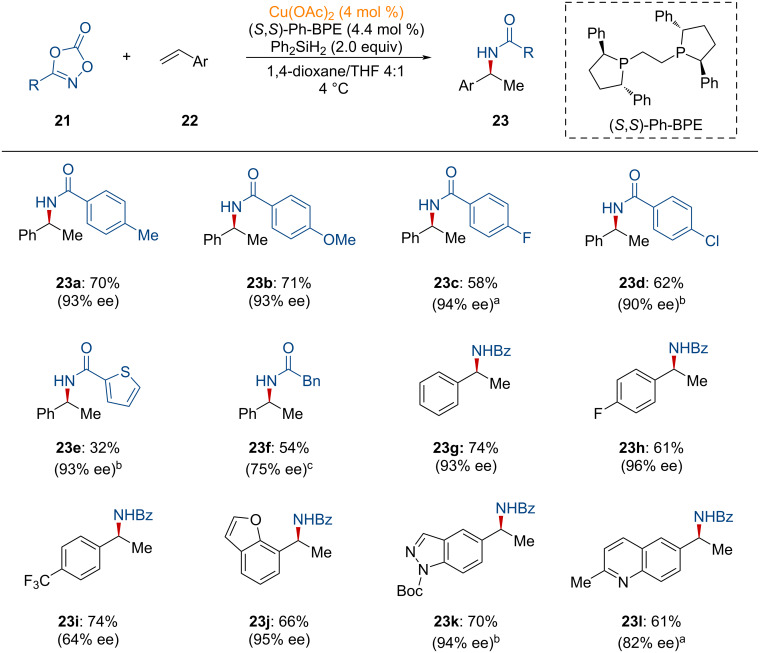
Copper-catalyzed asymmetric hydroamidation of vinylarenes. ^a^4 mol % + 2 mol % catalyst was used. ^b^4 mol % + 4 mol % catalyst was used. ^c^Slow addition of the amide electrophile solution.

As shown in Scheme 8, several dioxazolones containing electron-rich substituents were transformed into the desired products with excellent enantioselectivity (**23a** and **23b**). Otherwise, the electron-poor substituent-containing dioxazolones showed slightly diminished reactivity (**23c** and **23d**). In addition, poor reactivity was observed with dioxazolones bearing thiophene, implying that the undesired coordination of sulfur to copper reduces the reactivity (**23e**). Despite the reduced reactivity, excellent enantioselectivity was still maintained. Moreover, styrene derivatives bearing both electron-rich and electron-poor groups underwent the desired transformation with high yields and enantioselectivities (**23g** and **23h**). However, the styrene scaffold bearing a trifluoromethyl group showed reduced enantioselectivity (**23i**). Styrenes containing heterocyclic motifs such as a benzofuran, indazole, and quinoline were also shown to undergo the desired Markovnikov amidation with high efficiency (**23j**–**l**).

**Figure 7:**
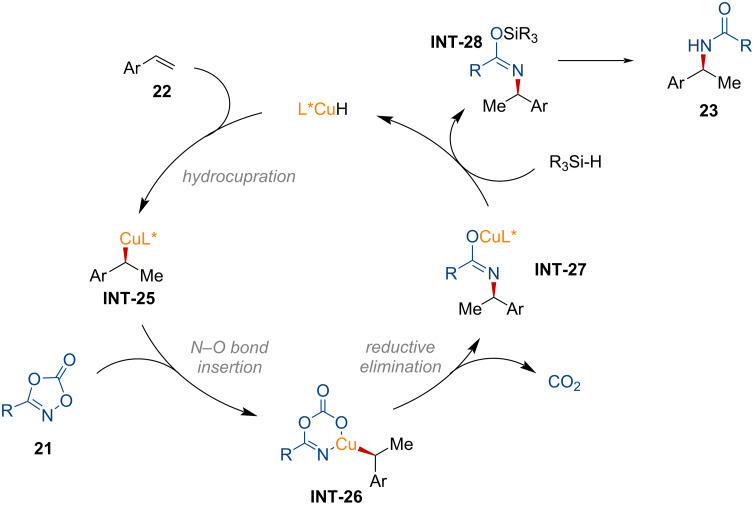
Proposed reaction mechanism for the copper-catalyzed hydroamidation of vinylarenes.

Several mechanistic experiments were performed to rationalize the reaction pathways. As shown in Figure 7, copper hydride, generated from a copper precatalyst and silane, undergoes the enantio-determining hydrocupration of the vinylarene, affording **INT**-**25** [25]. Next, oxidative insertion of **INT-25** into the N–O bond of the dioxazolone, forms **INT-26**, followed by decarboxylative reductive elimination to generate **INT-27**. Further incorporation of silane delivers the targeted amidated product upon protonation, while simultaneously regenerating the active copper hydride species.

#### 2.2 Hydroamidation of alkynes

**Scheme 9:**
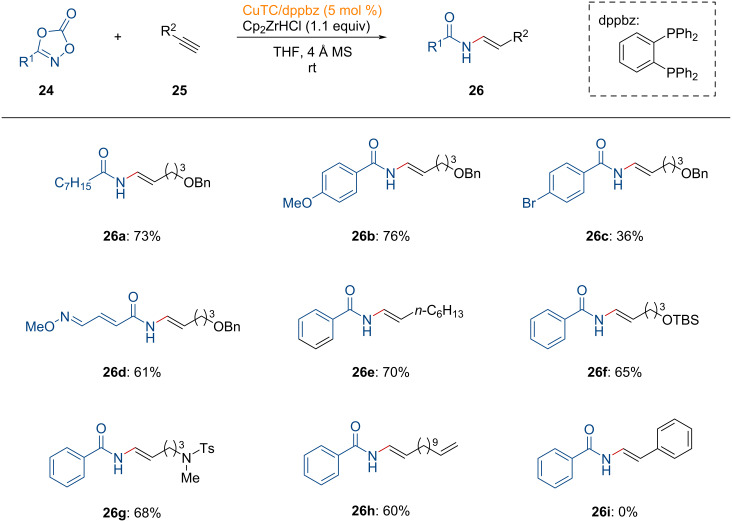
Copper-catalyzed anti-Markovnikov hydroamidation of alkynes.

In 2022, Sato and co-workers introduced a copper-catalyzed hydroamidation of alkynes **25** using dioxazolones **24** as amide sources (Scheme 9) [99].

**Figure 8:**
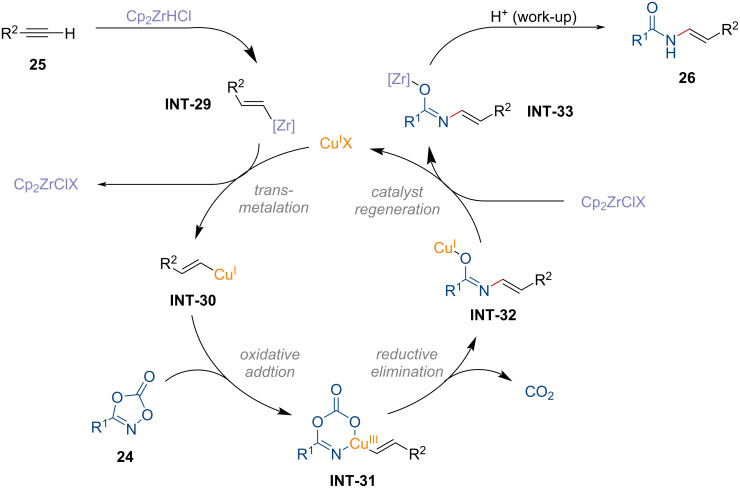
Proposed reaction mechanism for the copper-catalyzed amidation of alkynes.

A dioxazolone bearing a linear alkyl group was efficiently converted to the *N*-vinylamide **26a** in good yield. The observed regioselectivity followed the anti-Markovnikov fashion stemming from the regioselective hydrozirconation of the alkyne using Schwartz’s reagent [100]. Aryl substituents on the dioxazolone were tolerated, while a dioxazolone containing bromobenzene displayed lower reactivity (**26c**). The enamide **26d**, derived from lobatamide, was successfully produced without altering the stereochemistry of the oxime ether. Terminal alkynes with linear alkyl group, protected alcohol, and sulfonamide functionalities were well tolerated in this transformation (**26e**–**g**). Moreover, an olefin-containing terminal alkyne was suitable to afford product **26h**, demonstrating excellent chemoselectivity. However, the formation of **26i** was not observed under the standard reaction conditions. Instead, the decomposition of phenylacetylene was confirmed. Based on previous mechanistic insight from electrophilic amidation studies [101–102], the catalytic amidation of alkynes is proposed as shown in Figure 8.

First, the alkenylzirconium complex **INT-29**, formed through hydrozirconation of alkyne **25**, undergoes transmetalation with the copper catalyst, providing the vinyl copper intermediate **INT-30**. This intermediate then undergoes oxidative addition to the N–O bond of the dioxazolone to generate **INT-31**. Subsequently, decarboxylative reductive elimination occurs, forming the copper imidate **INT-32**. The active copper species is regenerated by the zirconium species, producing the formation of the desired product via the zirconium imidate **INT-33** after aqueous workup. Overall, this copper-catalyzed transformation requires a stoichiometric amount of zirconium species to achieve the anti-Markovnikov amidation.

### 3 Miscellaneous

#### 3.1 Synthesis of primary amides via the generation of copper–imidate radical intermediates

**Scheme 10:**
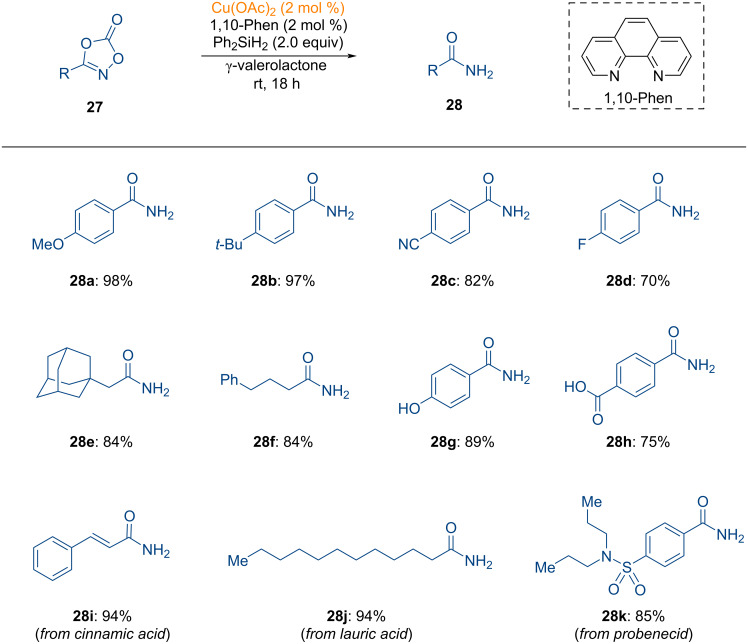
Copper-catalyzed preparation of primary amides through N–O bond reduction using reducing agent.

In a subsequent study, the research group of Son developed a method for the reduction of dioxazolones to synthesize primary amides under mild reducing conditions in copper catalysis (Scheme 10) [103].

**Figure 9:**
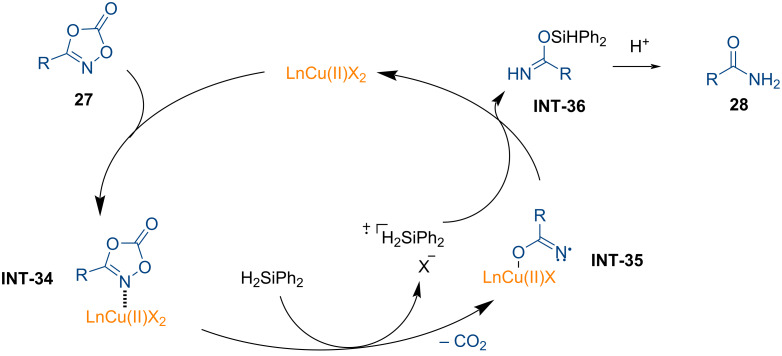
Proposed catalytic cycle for the copper-catalyzed reduction of dioxazolones.

The reaction was conducted using a catalytic amount of copper acetate and a phenanthroline ligand, with a stoichiometric amount of silane serving as the reductant. Both aryl- and alkyl-substituted dioxazolones proved to be compatible under the standard reaction conditions, yielding the desired primary amides **28a**–**f**. Notably, dioxazolones containing free -OH groups exhibited excellent functional group tolerance, leading to the formation of primary amides **28g** and **28h**. Moreover, biologically relevant scaffolds on dioxazolones were successfully transformed in this transformation (**28i**–**k**). As illustrated in Figure 9, a catalytic cycle was proposed for the copper-catalyzed synthesis of primary amides.

Dioxazolone first binds to the copper catalyst, forming the dioxazolone-copper complex (**INT-34**). The silane reductant then reduces **INT-34**, forming the copper imidate radical species **INT-35**, furnishing the formation of primary amides **28** via intermediate **INT-36**. The involvement of a radical intermediate was suggested by experiments using TEMPO as a radical scavenger.

## Conclusion

This review provides an overview of the recent copper-catalyzed and/or -promoted transformations of dioxazolones, describing examples of C(sp^2^)–N, C(sp^3^)–N, S–N, P–N, and N–H bond-forming reactions. Several studies have proposed copper nitrenoid intermediates originating from dioxazolones, involving an *N*-acyl nitrene transfer or hydrogen atom transfer process, representing creative synthetic solutions that were previously unachievable using conventional approaches. Despite the synthetic strategies to generate the copper nitrenoid intermediates are gaining more attention [104–105], several limitations still remain. First, multiple steps are required to prepare dioxazolones, synthesized conventionally from carboxylic acids or acid chlorides over two steps. Moreover, copper-catalyzed asymmetric C(sp^3^)–N bond-forming transformations are still underexplored except for elegant studies by the research groups of Buchwald [98] and Chang [74]. Given the versatility and sustainability of the copper-catalyzed transformation of dioxazolones, further investigations for late-stage functionalizations of complex scaffolds such as peptides, drug molecules, and natural products containing unprotected free OH and NH groups are anticipated.

## Data Availability

Data sharing is not applicable as no new data was generated or analyzed in this study.
